# User Real Comments Incentive Mechanism Based on Blockchain in E-Commerce Transactions—A Tripartite Evolutionary Game Analysis

**DOI:** 10.3390/e26121005

**Published:** 2024-11-22

**Authors:** Chengyi Le, Ran Zheng, Ting Lu, Yu Chen

**Affiliations:** 1 School of Business, Ningbo University, Ningbo 315211, China; 2 School of Economics and Management, East China Jiaotong University, Nanchang 330013, China; tsangryann@gmail.com (R.Z.); 13268589702@163.com (Y.C.); 3 Institution of Education, Capital Normal University, Beijing 100089, China; luting_88@126.com; 4 School of Humanities and Arts, Nanchang Institute of Technology, Nanchang 330099, China

**Keywords:** blockchain, e-commerce transactions, real comments, smart contracts, tripartite evolution game

## Abstract

In response to the widespread issue of fake comments on e-commerce platforms, this study aims to analyze and propose a blockchain-based solution to incentivize authentic user feedback and reduce the prevalence of fraudulent reviews. Specifically, this paper constructs a tripartite evolutionary game model between sellers, buyers, and e-commerce platforms to study the real comment mechanism of blockchain. The strategy evolution under different incentive factors is simulated using replication dynamic equation analysis and Matlab software simulation. The study found that introducing smart contracts and “tokens” for incentives not only increased incentives for real comments but also reduced the negative experiences caused by “speculative” sellers, thereby influencing buyers to opt for authentic reviews. By structuring interactions through blockchain, the mechanism helped lower informational entropy thus reducing disorder and unpredictability in buyer and seller behavior and contributing to system stability. Further, by increasing penalties for dishonest behavior under the “credit on the chain” system, the platform lowered entropy in the system by promoting trust and reducing fraudulent activities. The real comment mechanism based on blockchain proposed in this paper can effectively enhance the order and transparency within the comment ecosystem. These findings contribute to theory and practice by providing strategic insights for e-commerce platforms to encourage genuine feedback, reduce informational entropy, and mitigate fake comments, ultimately fostering a more reliable online marketplace.

## 1. Introduction

In the Internet era, comments on goods posted by users after shopping on e-commerce websites have become the main contents generated by network users [1]. These product purchase comments not only have a significant impact on potential consumers [2] but also serve as valuable feedback for merchants to improve on their products and enhance the user experience. However, the rise in fake comments—reviews that do not accurately reflect the user’s true opinion—has introduced significant challenges in e-commerce, particularly affecting consumer trust and the overall reliability of digital markets. This prevalence of fake comments can skew purchasing decisions, erode trust in online platforms, and undermine the competitive fairness of the digital marketplace. Driven by commercial interests, some sellers employ consumers and posters to publish deceptive fake comments, leading to the proliferation of fake comments. This phenomenon increases the informational entropy in the system, creating disorder and unpredictability in the e-commerce environment, which reduces market efficiency and hinders the healthy development of e-commerce [3]. Moreover, an excess of fake comments can drown out valuable real comments, which express genuine user experiences with goods and services. This leads to a “bad money drives out good” effect, causing users to gradually lose the motivation to provide real feedback. Consequently, how to effectively address fake comments and ensure genuine interactions on e-commerce platforms has become a critical challenge, capturing the attention of researchers and practitioners alike [4,5].

Existing methods for combating fake reviews, such as third-party verification and machine learning-based detection, have been limited in their effectiveness. Traditional systems struggle with identifying strategic fraud such as ballot-stuffing or camouflage attacks, which manipulate the review system in more subtle ways [6]. Blockchain, however, offers a promising solution to these challenges by providing transparency, traceability, and tamper-proofing features that can help ensure the authenticity of reviews. Blockchain-based systems ensure that reviews cannot be altered retroactively, making them resistant to manipulations like bad-mouthing and whitewashing, which are common in traditional review systems [7]. Additionally, blockchain’s decentralized nature addresses privacy concerns, allowing users to participate honestly in review systems without fear of losing anonymity [8].

“Fake comments” was first proposed by Jindal and Liu (2008), who observed that fake online comments were widespread. Fake comments refer to online feedback that is inconsistent with the true evaluation of a product or service; they are false, deceptive, and forged [9]. The main motivations behind fake comments are promotion or slander, which disrupt the accuracy and reliability of product/service descriptions. Real comments, on the other hand, provide genuine experiences and evaluations, helping consumers accurately identify high-quality products and enhancing trust in the platform. Currently, most research on combating fake comments on e-commerce platforms focuses on detecting, identifying, punishing, and eliminating fake comments. Existing studies have explored the motivations behind fake comments and their influence on consumers’ purchasing decisions [10], the identification and detection of fake comments [11,12], and the methods to prevent and address fake comments [13]. While these efforts have had some success in reducing the occurrence of fake comments, fake comments remain widespread, harmful, and difficult to identify manually [14]. Additionally, the detection methods for fake comments have limitations in efficiency and accuracy. This reactive approach of “plugging leaks” does not sufficiently reduce fake comments or promote real ones, failing to decrease the informational entropy caused by deceptive behaviors. This study, therefore, addresses the following research problem: How can blockchain technology be used to reduce the prevalence of fake comments and promote authentic feedback in e-commerce transactions?

To tackle this issue, this research aims to analyze and propose a blockchain-based solution to address fake comments and foster genuine interactions in e-commerce. There is limited research on how to motivate users to provide real comments. Some scholars have proposed mechanisms based on prior probabilities from historical comments to encourage real feedback. For instance, Richard and Nolan proposed the “peer prediction method” [15], while Witkowski and Parkes introduced the “private-prior peer prediction mechanism” and Kamar and Horvitz proposed the “consensus prediction rule” [16,17]. However, these mechanisms primarily rely on inductive reasoning based on unknown information, which can lead to misjudgment when a randomly selected reviewer happens to be a fake reviewer. Blockchain, as a new generation of technical support, offers a promising approach to reducing informational entropy within the e-commerce ecosystem due to its decentralization and traceability features [18]. Several scholars have started exploring how blockchain can be utilized to curb fake comments and stimulate real ones. Liu proposed a blockchain-based e-commerce review model, incorporating a weighted rating system based on reviews from traditional e-commerce stores [19]. Zhou et al. suggested using “tokens” in blockchain-based e-commerce to encourage consumers to participate in shaping authentic word-of-mouth feedback, motivating users to leave real comments and converting them into supervisory nodes [20]. Davide Carboni built a reputation model based on blockchain, using a decentralized and distributed feedback management system to reward consumers with electronic vouchers in exchange for honest comments [21]. However, this model primarily addresses buyer behavior and does not consider the dominant role that sellers play in generating fake comments.

This study sets forth several primary objectives as follows: (1) to design a blockchain-based real comment mechanism aimed at promoting genuine user feedback and reducing fake reviews; (2) to investigate the specific incentives and penalties that would effectively encourage honest interactions between buyers and sellers; and (3) to model the strategic interactions among buyers, sellers, and e-commerce platforms through a tripartite evolutionary game framework.

The key contributions of this study to both theory and practice are as follows: First, from the perspective of promoting real comments, it explores how to incentivize users to provide genuine feedback by offering rewards. Second, it introduces theoretical innovations by addressing the shortcomings of existing comment mechanisms and proposing a blockchain-based real comment system. Third, it applies the tripartite evolutionary game model to analyze the dynamic behavior evolution of sellers, buyers, and platforms, highlighting how the blockchain-based real comment mechanism can help reduce informational entropy in the e-commerce ecosystem and promote honest behavior. Finally, by focusing on the practical applications of blockchain technology, this research provides actionable insights for e-commerce platforms seeking to foster trust and transparency in user feedback, offering strategic guidance for managing user-generated content in a more reliable manner.

## 2. Incentive Mechanism for User Real Comments of E-Commerce Transactions

Blockchain has the characteristics of decentralization, traceability, and non-tampering technology, which can effectively regulate the transaction behavior of both buyers and sellers. The use of “tokens” or “vouchers” can encourage buyers to leave real comments and motivate sellers to operate in good faith. These blockchain features will enhance trust by providing incentives for honest behavior and penalties for dishonest actions, such as submitting fake reviews. By using blockchain’s decentralized structure, the system ensures transparency, preventing the manipulation of reviews and improving the overall reliability of the feedback system [22].

**Figure 1:**
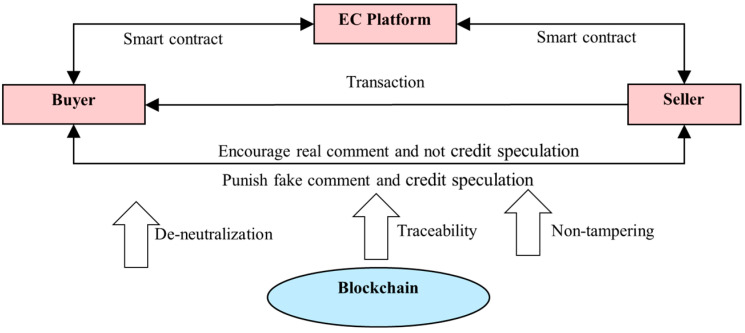
Incentive mechanism of user real comments based on blockchain.

Building on Carboni’s reputation model and focusing on incentivizing real comments, this paper proposes a blockchain-based mechanism to encourage honest behavior in e-commerce transactions [21]. This mechanism is designed to increase the rewards for honesty and the penalties for dishonesty among both buyers and sellers. Blockchain’s inherent qualities—such as decentralization and immutability—ensure that review data are transparent and cannot be tampered with after submission. This provides a more trustworthy system compared to traditional review mechanisms, which are often vulnerable to manipulation [23] (Figure 1).

In this mechanism, the platform signs a smart contract with buyers, offering incentives such as “access cards”, vouchers, or other rewards to those who promise to leave real reviews [24]. These rewards are intended to motivate buyers to engage in the review process. At the same time, blockchain technology is used to detect fake reviews by cross-referencing transaction data and identifying suspicious patterns. If a buyer submits a fake review, penalties will be automatically applied, ensuring that dishonest behavior is detected and penalized. This automated process helps maintain the integrity of the review system [25].

Similarly, sellers who maintain integrity will be rewarded with positive reputation scores, enhancing their credibility and trustworthiness on the platform. On the other hand, sellers engaging in “credit speculation” (artificially inflating reviews to improve their reputation) will be penalized. These penalties are also recorded on the blockchain, making the actions of both buyers and sellers traceable and irreversible. The use of blockchain’s “time-stamping” feature ensures that all review and transaction data are securely recorded, preventing any manipulation or tampering. This approach helps to create a more transparent and trustworthy marketplace by ensuring that both parties are incentivized to act honestly [26].

## 3. Model Assumptions and Construction

### 3.1. Model Parameter Setting

In the e-commerce transaction process, three parties are involved—the seller, the buyer, and the e-commerce platform—and their earnings are influenced by two distinct strategic choices. Referring to the existing research on blockchain game mechanism, and upon consulting experts in the field of e-commerce, we established a tripartite evolutionary game model of the sellers, buyers, and e-commerce platform, the specific parameters of the which are shown in Table 1 [27,28].

In Table 1, “O_2_” represents the total cost that e-commerce platforms need to pay to incentivize other game participants after adopting the decision of blockchain incentives. Although “In” and “En” are both benefits brought to buyers by e-commerce platforms, there are differences between the two. “In” is a token reward unique to the blockchain incentive mechanism, while “En” is a reward content that is different from the blockchain, such as user reputation level, coupons, etc.

In the process of e-commerce transactions, the cost H of the seller’s honest operation is smaller than the cost of the seller’s “speculation” operation C_1_, C_1_ > H. The income of the seller’s “speculation” operation is greater than the income of the seller’s honest operation GI, GI > Pr. The probability of the seller’s “speculation” being verified is ψ, 0 ≤ ψ ≤ 1. The platform’s penalty coefficient for “speculation” sellers is λ, 0 ≤ λ ≤ 1, and the greater the income of “speculation”, the greater the penalty coefficient. The cost T of buyers making real reviews is greater than the cost of buyers making false reviews T_2_, T_2_ > T. If the buyer gives high-quality praise, including the number of words in the review, the form of the review, and the content of the review within the specified time, the r value is greaterincreases, 0 ≤ r ≤ 1. The probability that a buyer will make a fake review and be verified by the platform is α, 0 ≤ α ≤ 1.

### 3.2. Model Assumptions

To build a tripartite game model and analyze the strategies and stability of the equilibrium points of sellers, buyers, and e-commerce platforms, as well as the influence of and relationships between various elements, the following hypotheses are made:

**Assumption** **1.** *In the process of e-commerce, transactions among the buyer, the seller, and the e-commerce platform are bound to be rational and will constantly optimize their own strategy selection in the learning process, as well as choose the strategy selection corresponding to the maximization of their own payment function.*

**Assumption** **2.** *Assume that all e-commerce platforms will verify and punish sellers of fake comments and “stir-frying”.*

**Assumption** **3.** *The quality of goods and services provided by the seller is relatively poor.*

**Assumption** **4.** *The sellers’ strategies include A1 “not credit speculation” and A2 “credit speculation”. Among the sellers, the proportion of sellers who choose A1 is X (0 ≤ X ≤ 1), and the proportion of sellers who choose A2 is 1 − X. Buyers’ strategies can be “B1 real comments” or “B2 fake comments”. Among the buyers, the proportion of buyers who choose B1 is Y (0 ≤ Y ≤ 1), and the proportion of buyers who choose B2 is 1 − Y. At the same time, e-commerce platforms can choose the following two strategies: “W1 encourages honest behavior” and “W2 does not encourage honest behaviors”. Among them, the proportion of e-commerce platforms that choose “W1” is W (0 ≤ W ≤ 1), and the proportion of e-commerce platforms that choose “W2” is 1 − W.*

### 3.3. Model Construction

According to the above assumptions, in the process of e-commerce trading, the evolutionary game revenue matrix among the seller, buyer, and e-commerce platform in e-commerce trading is presented in the following sections.

## 4. Tripartite Evolutionary Game Analysis

### 4.1. Replication Dynamic Equation Analysis

In the process of e-commerce transactions, the strategic choices of the seller, buyer, and e-commerce platform interact and influence each other, and the three parties constantly adjust their strategic choices to achieve the maximum expected benefits [29]. According to the benefits of the three parties’ different strategies, the dynamic replication equations of the three parties’ respective evolutionary games are established to solve the forming conditions and process of the evolutionary stability strategy.

According to the income matrix of e-commerce platform, buyer, and seller in Table 2, the expected income of the seller choosing an honest operation is as follows:

The seller’s expected income from a “stir-fry” operation is as follows:

(1) $$V_{a_{1}}=\mathrm{WY}\left(\mathrm{Pr}+En_{2}-H\right)+W\left(1-Y\right)\left(\mathrm{Pr}+En_{2}-H\right)+\left(1-W\right)Y\left(\mathrm{Pr}-H\right)+\left(1-W\right)\left(1-Y\right)\left(\mathrm{Pr}-H\right)=\mathrm{WE}n_{2}+P-H$$

(2) $$\begin{array}{l} V_{a_{2}}=\mathrm{WY}(\mathrm{GI}-C_{1}-C_{2}-\psi \lambda P)+(1-Y)W(\mathrm{GI}-C_{1}-\psi \lambda P)+Y(1-W)(\mathrm{GI}-C_{1}-C_{2}-\psi \lambda P)+(1-Y)(1-W)(\mathrm{GI}-\\ C_{1}-\psi \lambda P)=-YC_{2}+\mathrm{GI}-C_{1}-\psi \lambda P \end{array}$$

The average revenue of the seller group is as follows:

(3) $$\bar{U}=YV_{a1}+(1-Y)V_{a2}$$

The replication dynamic equation chosen by the seller’s strategy is as in the following equation:

(4) $$F(X)=X\left(1-X\right)\left(V_{a_{1}}-V_{a_{2}}\right)=X\left(1-X\right)\left(\mathrm{WE}n_{2}+\mathrm{Pr}-H+YC_{2}-\mathrm{GI}+C_{1}+\psi \lambda P\right)$$

Similarly, the expected earnings of buyers choosing to make real comments and fake comments are Ub_1_ and Ub_2_, as follows:

(5) $$U_{b_{1}}=\mathrm{WEn}+\mathrm{XTh}+E+\mathrm{In}-T-\mathrm{Th}$$

(6) $$U_{b_{2}}=(D+\mathrm{rF})-T_{2}-\mathrm{aN}$$

The replication dynamic equation of buyer group strategy selection is as follows:

(7) $$F(Y)=Y(1-Y)[\mathrm{WEn}+\mathrm{XTh}+E+\mathrm{In}-T-\mathrm{Th}-\mathrm{rF}+T_{2}+\mathrm{aN}]$$

The expected returns of the platform choosing to motivate real comments and not to motivate real comments are Ωq_1_ and Ωq_2_, as in the following equations:

(8) $$\Omega_{q_{1}}=I-O_{1}-O_{2}$$

(9) $$\Omega_{q_{2}}=-L-O_{1}$$

The replication dynamic equation of platform strategy selection is as follows:

(10) $$F(W)=W(1-W)(I-O_{2}+L)$$

To sum up, the replicative dynamic equations derived from the tripartite evolutionary game are as follows:

$$F(X)=X(1-X)(\mathrm{WE}n_{2}+\mathrm{Pr}-H+YC_{2}-\mathrm{GI}+C_{1}+\psi \lambda P)$$

$$F(Y)=Y\left(1-Y\right)\left(\mathrm{WEn}+\mathrm{XTh}+E+\mathrm{In}-T-\mathrm{Th}-\mathrm{rF}+T_{2}+\alpha N\right)$$

$$F(W)=W(1-W)(I-O_{2}+L)$$

### 4.2. Evolutionary Stability Strategy Analysis

Let F(X) = 0, F(Y) = 0, F(W) = 0, and the possible replication dynamic stability points are J1(0, 0, 0), J2(1, 0, 0), J3(0, 1, 0), J4(0, 0, 1), J5(1, 1, 0), J6(1, 0, 1), J7(0, 1, 1), J8(1, 1, 1), and J9(X*, Y*, W*), where J9(X*, Y*, W*) is the saddle point. Based on Ritzberger and Weibull research conclusion, the evolutionary stable strategy in a multi-population evolutionary game—whether for pure strategy Nash equilibrium or asymmetric mixed strategy equilibrium—will inevitably fail to reach a stable evolutionary state, so we only need to analyze J1(0, 0, 0), J2(1, 0, 0), J3(0, 1, 0), J4(0, 0, 1),J5(1, 1, 0), J6(1, 0, 1), J7(0, 1, 1), and J8(1, 1, 1). Lyapunov’s first method is used for the stability of the equilibrium points in tripartite evolutionary games. The stability of each equilibrium point is as follows:

$$J=\left(\begin{array}{ccc} \frac{\partial F(X)}{\partial x} & \frac{\partial F(X)}{\partial y} & \frac{\partial F(X)}{\partial w}\\ \frac{\partial F(Y)}{\partial x} & \frac{\partial F(Y)}{\partial y} & \frac{\partial F(Y)}{\partial w}\\ \frac{\partial F(W)}{\partial x} & \frac{\partial F(W)}{\partial y} & \frac{\partial F(W)}{\partial w} \end{array}\right)$$

The corresponding Jacobian matrix and eigenvalues of the matrix can be obtained by substituting the equilibrium points of the system with the Jacobian matrix of the 3D dynamical system. According to Lyapunov’s first method, when all the eigenvalues of the Jacobi matrix are not positive, the strategy subset is the evolutionary stable point of the 3D replication dynamical system. By substituting eight policy subsets into the Jacobian matrix J, the Jacobian matrix and eigenvalues of the 3D replication dynamical system corresponding to all policy subsets can be obtained, as shown in Table 3.

It can be seen from Table 3 that the positivity and negativity of the three eigenvalues of the Jacobian matrix are related to the benefits of three different strategies. When λ1, λ2, and λ3 are all negative, this point is the evolutionary stability point of the system. Therefore, the stability analysis of the eight strategy set points involves 10 groups of benefit comparisons. The following eight cases of Jacobian matrix eigenvalues are discussed.

① Case 1: When $(\mathrm{Pr}-H)>(\mathrm{GI}-C_{1}-C_{2}-\psi \lambda P),\;(E-T-\mathrm{Th})>(\mathrm{rF}-T_{2}-\alpha N),\;(I-O_{2})>\left(-L\right)$, J8(1, 1, 0) is the evolutionary stability point of the system, that is, sellers choose to operate in good faith, buyers choose to make real comments, and e-commerce platforms choose to encourage honest behaviors.
② Case 2: When $(\mathrm{Pr}-H)>(\mathrm{GI}-C_{1}-C_{2}-\psi \lambda P),\;(E-T-\mathrm{Th})>(\mathrm{rF}-T_{2}-\alpha N),\;(I-O_{2})<\left(-L\right)$, J5(1, 1, 0) is the evolutionary stability point of the system. At this point, the seller chooses to operate in good faith, the buyer chooses to make real comments, and the e-commerce platform chooses not to encourage honest behavior.
③ Case 3: When $(\mathrm{Pr}-H)>(\mathrm{GI}-C_{1}-\psi \lambda P),\;(E-T)<(\mathrm{rF}-T_{2}-\alpha N),\;(I-O_{3})>\left(-L\right)$, The evolution of the system stability and the evolution trend are related to the parameter En (incentives for honest buyer), In (blockchain comment mechanism for buyers real comment incentives), En2 (incentives for the honest sellers), C2 (the seller should pay the extra cost of “credit speculation” when buyers choose real comment), and Th (harassment and other negative experiences to buyers). And it is divided into the following three situations:
  - When $\mathrm{En}+\mathrm{In}<[\mathrm{rF}-T_{2}-K-\alpha (N+\mathrm{Ne})]-(E-T-\mathrm{Th})$, that is, $(\mathrm{En}+\mathrm{In}+E-T)-[\mathrm{rF}-T_{2}-K-\alpha (N+\mathrm{Ne})]<0$, and if $En_{2}-C_{2}<(\mathrm{GI}-C_{1}-C_{2}-\psi \lambda P)-(\mathrm{Pr}-H)$, then J4(0, 0, 1) is the evolutionary stability point of the system;
  - When $\mathrm{En}+\mathrm{In}+\mathrm{Th}<[\mathrm{rF}-T_{2}-K-\alpha (N+\mathrm{Ne})]-(E-T-\mathrm{Th})$, if $En_{2}-C_{2}>(\mathrm{GI}-C1-C2-\psi \lambda P)-(\mathrm{Pr}-H)$, then J6(1, 0, 1) is the evolutionary stability point of the system;
  - When $\mathrm{En}+\mathrm{In}+\mathrm{Th}>[\mathrm{rF}-T_{2}-K-\alpha (N+\mathrm{Ne})]-(E-T-\mathrm{Th})$, that is, $(\mathrm{En}+\mathrm{In}+E-T)-[\mathrm{rF}-T_{2}-\alpha N]<0$, then J8(1, 1, 1) is the evolutionary stability point of the system.
④ Case 4: When $(\mathrm{Pr}-H)>(\mathrm{GI}-C_{1}-C_{2}-\psi \lambda P),\;(E-T-\mathrm{Th})<(\mathrm{rF}-T_{2}-\alpha N),\;(I-O_{2})<\left(-L\right)$, the evolution of the system stability and the evolution trend are related to the parameters In, C2, and Th. And this can be divided into the following three situations:
  - When $\mathrm{In}<[\mathrm{rF}-T_{2}-\alpha N]-(E-T-\mathrm{Th})$, that is, $(\mathrm{In}+E-T-\mathrm{Th})-(\mathrm{rF}-T_{2}-\alpha N)<0$, and if $C_{2}>(\mathrm{Pr}-H)-(\mathrm{GI}-C_{1}-C_{2}-\psi \lambda P)$, then J1(0, 0, 0) is the evolutionary stability point of the system;
  - When $\mathrm{In}+\mathrm{Th}<[\mathrm{rF}-T_{2}-\alpha N]-(E-T-\mathrm{Th})$, that is, $(\mathrm{In}+E-T)-(\mathrm{rF}-T_{2}-\alpha N)<0$, and if $C_{2}<(\mathrm{Pr}-H)-(\mathrm{GI}-C_{1}-C_{2}-\psi \lambda P)$, then J2(1, 0, 0) is the evolutionary stability point of the system;
  - When $\mathrm{In}+\mathrm{Th}>(\mathrm{rF}-T_{2}-\alpha N)-(E-T-\mathrm{Th})$, that is, $(\mathrm{rF}-T_{2}-\alpha N)-(\mathrm{In}+E-T)<0$, then J5(1, 1, 0) is the evolutionary stability point of the system.
⑤ Case 5: When $(\mathrm{Pr}-H)<(\mathrm{GI}-C_{1}-C_{2}-\psi \lambda P),\;(E-T-\mathrm{Th})>(\mathrm{rF}-T_{2}-\alpha N),\;(I-O_{2})>\left(-L\right)$, the evolution of the system stability and the evolution trend are related to the parameter En2. Specifically, when $(En_{2}+\mathrm{Pr}-H)-(\mathrm{GI}-C_{1}-C_{2}-\psi \lambda P)<0$, J7(0, 1, 1) is the evolutionary stability point of the system. On the contrary, J8(1, 1, 1) is the evolutionary stable equilibrium strategy of the system.
⑥ Case 6: When $(\mathrm{Pr}-H)<(\mathrm{GI}-C_{1}-C_{2}-\psi \lambda P),\;(E-T-\mathrm{Th})>(\mathrm{rF}-T_{2}-\alpha N),\;(I-O_{2})<\left(-L\right)$, J3(0, 1, 0) is the evolutionary stability point of the system.
⑦ Case 7: When $(\mathrm{Pr}-H)<(\mathrm{GI}-C_{1}-C_{2}-\psi \lambda P),\;(E-T-\mathrm{Th})<(\mathrm{rF}-T_{2}-\alpha N),\;(I-O_{2})>(-L)$, the evolution of the system stability and the evolution trend are related to the parameters En and Th. And this can be divided into the following four situations to:
  - When $\mathrm{En}+\mathrm{In}<(\mathrm{rF}-T_{2}-K-\alpha N)-(E-T-\mathrm{Th})$, if $En_{2}-C_{2}<(\mathrm{GI}-C_{1}-C_{2}-\psi \lambda P)-(\mathrm{Pr}-H)$, then J4(0, 0, 1) is the stable equilibrium strategy of system evolution;
  - When $\mathrm{En}+\mathrm{In}+\mathrm{Th}<(\mathrm{rF}-T_{2}-\alpha N)-(E-T-\mathrm{Th})$, if $En_{2}-C_{2}>(\mathrm{GI}-C_{1}-C_{2}-\psi \lambda P)-(\mathrm{Pr}-H)$, then J6(1, 0, 1) is the stable equilibrium strategy of system evolution;
  - When $\mathrm{En}+\mathrm{In}>(\mathrm{rF}-T_{2}-\alpha N)-(E-T-\mathrm{Th})$, if $En_{2}<(\mathrm{GI}-C_{1}-C_{2}-\psi \lambda P)-(\mathrm{Pr}-H)$, then J7(0, 1, 1) is the stable equilibrium strategy of system evolution;
  - When $\mathrm{En}+\mathrm{In}+\mathrm{Th}>(\mathrm{rF}-T_{2}-\alpha N)-(E-T-\mathrm{Th})$, if $En_{2}>(\mathrm{GI}-C_{1}-C_{2}-\psi \lambda P)-(\mathrm{Pr}-H)$, then J8(1, 1, 1) is the stable equilibrium strategy of system evolution.
⑧ Case 8: When (Pr − H) < (GI − C1 − C2 − ψλP), (E − T − Th) < (rF − T2 − αN), (I − O2) < (−L), if $\mathrm{In}<(\mathrm{rF}-T_{2}-\alpha N)-(E-T-\mathrm{Th})$, J1(0, 0, 0) is the evolutionary stability point of the system. On the contrary, J3(0,1,0) is the evolutionary stable equilibrium strategy of the system.

## 5. Simulation

To further analyze the mechanism and specific evolution trend of the influence of the strategic choice of the seller, buyer, and e-commerce platform, this study will set the model parameters reasonably according to the reality of e-commerce transactions [30], as shown in Table 4 [31]. MATLAB2016a software is used to simulate the evolution trend of the tripartite evolutionary game system. The initial willingness of the three parties to participate is 0.5.

**Figure 2:**
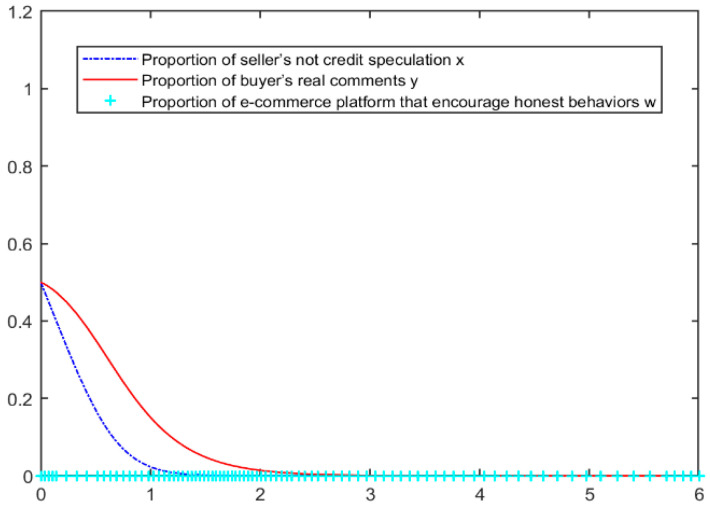
Evolution simulation results by initialization parameter—without blockchain.

**Figure 3:**
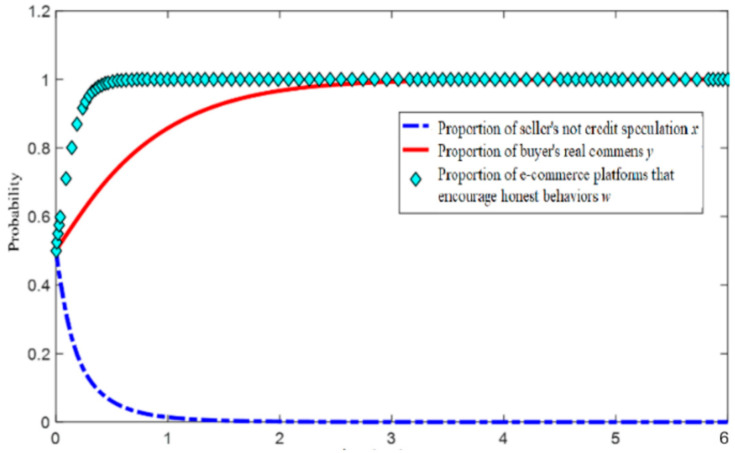
Evolution simulation results by initialization parameter—with blockchain.

According to the initial parameters, we stimulated two situations when the blockchain comment mechanism is not added and is added, MATLAB simulations results are shown in Figure 2 and Figure 3. we can see that when the blockchain mechanism is not added, even if the initial proportion of buyers and sellers reaches 50%, the equilibrium point of the game between the two sides will slowly fall to (0,0), and the comment environment will gradually become chaotic. Among them, the seller will act before the buyer, driving the decline of the buyer’s comment behavior.

After the introduction of the blockchain real comment mechanism, the evolution stability point of the system is (0, 1, 1), that is, sellers engage in credit speculation, buyers choose to make real comments, and e-commerce platforms encourage honest behavior. In this state, the e-commerce platform needs to continuously invest money to maintain the buyer’s willingness to make real comments, but the seller continues to be in a balanced strategy of zero and will continue to try to encourage the buyer to make fake comments. The evolution results of the initial parameters are roughly consistent with the actual situation of the current sellers’ credit speculation operation, most buyers’ real comment, e-commerce platforms constantly improving their platform management and evaluation models and curbing fake comments [32,33]. For sellers, dishonesty is still profitable, and the parameters of the three parties in the comment mechanism need to be further adjusted.

According to the above analysis, the research hopes that the tripartite evolutionary game can be stable at the strategic gathering point (1, 1, 1), i.e., (sellers do not engage in credit speculation, buyers choose to make real comments, and e-commerce platforms encourage honest behavior), which needs to meet one of the following conditions:

Condition 1: $\left(\mathrm{Pr}-H\right)>\left(\mathrm{GI}-C_{1}-C_{2}-\varphi \lambda P\right),\;\left(E-T-\mathrm{Th}\right)>\left(\mathrm{rF}-T_{2}-\alpha N\right),\;\mathrm{and}\;\left(I-O_{2}\right)>\left(-L\right)$;

Condition 2: $\left(\mathrm{Pr}-H\right)>\left(\mathrm{GI}-C_{1}-C_{2}-\varphi \lambda P\right),\;\left(E-T-\mathrm{Th}\right)<\left(\mathrm{rF}-T_{2}-\alpha N\right),\;\left(I-O_{2}\right)>\left(-L\right),\;\mathrm{and}\;E+\mathrm{In}+\mathrm{Th}>\left(\mathrm{rF}-T_{2}-\alpha N\right)-\left(E-T-\mathrm{Th}\right)$;

Condition 3: $\left(\mathrm{Pr}-H\right)<\left(\mathrm{GI}-C_{1}-C_{2}-\varphi \lambda P\right),\;\left(E-T-\mathrm{Th}\right)>\left(\mathrm{rF}-T_{2}-\alpha N\right),\;\left(I-O_{2}\right)>\left(-L\right),$ $\mathrm{and}\;En_{2}>\left(\mathrm{GI}-C_{1}-C_{2}-\varphi \lambda P\right)-\left(\mathrm{Pr}-H\right)$;

Condition 4: (Pr − H) < (GI − C_1_ − C_2_ − φλP), (E − T − Th) < (rF − T_2_ − αN), (I − O_2_) > (−L), and E + In + Th > (rF − T_2_ − αN) − (E − T − Th), En2 > (GI − C_1_ − C_2_ − φλP) − (Pr − H).

### 5.1. Simulation of Incentive of Honest Seller on E-Commerce Platform

**Figure 4:**
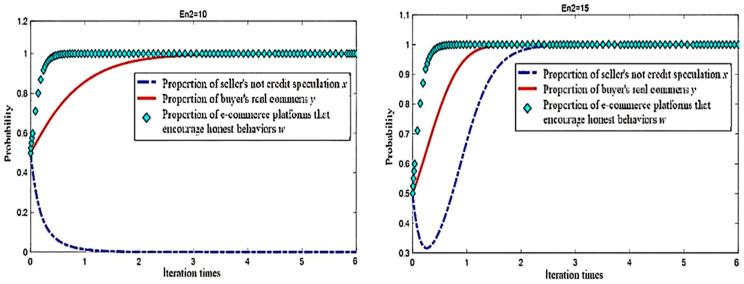
Simulation results with different En2.

In the analysis of an electric business platform for “no credit speculation”, the seller’s integrity level changes during the evolutionary game process, while the influence of the other parameters remain unchanged under the condition where the electric business platform for seller incentives for integrity (En2) are 10 and 15; the simulation results of the evolution of the replication dynamic equations over time are shown in Figure 4.

In the process of system evolution to a stable point, when the other conditions remain unchanged, the increase in incentives provided by the e-commerce platforms for sellers who “do not engage in credit speculation” accelerates the evolution of the sellers’ choice not to engage in “credit speculation”. With the increase in En2, the probability of sellers choosing “do not engage in credit speculation” increases (see Figure 3).

**Figure 5:**
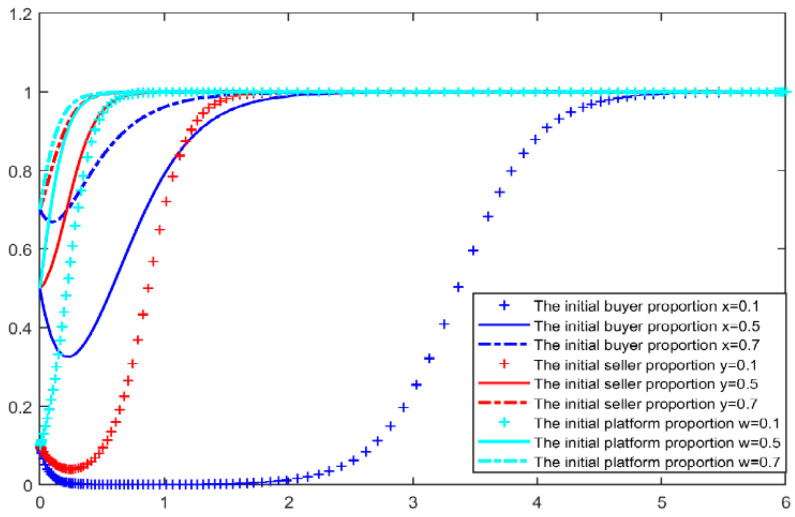
Evolution simulation results when En2 = 15.

When En2 reaches a certain level, no matter how the other conditions change, as long as condition 1 is met, the system will eventually converge to (1, 1, 1), even if the initial willingness of the seller to choose “not to engage in credit speculation” is very low, as low as 0.1, as shown in Figure 5. Therefore, e-commerce platforms should strengthen the incentives for sellers who “do not engage in credit speculation”, to increase the benefits of and guide sellers’ honest operations [34].

It is worth noting that different initial proportions have little impact on the evolution speed of e-commerce platforms, and platforms rapidly evolve to adopt incentive policies. This demonstrates that the platform’s behavior reaches a stable low-entropy state quickly, minimizing the unpredictability in its strategic evolution. However, sellers are highly sensitive to different incentives and initial proportions. When both the buyer’s and platform’s incentive levels are low, sellers will only begin to evolve toward honest behavior once the buyers and platforms approach a stable point. In this case, the entropy of the system remains high due to the delayed response from sellers. Conversely, when the incentive levels of buyers and platforms are higher, sellers react more quickly to changes in buyer and platform behavior, reducing the overall system entropy more rapidly by converging toward honest strategies. This demonstrates how increased incentives can lower the disorder in the seller’s decision-making process, leading to a more efficient and predictable evolution of the ecosystem. Similar conclusions have been drawn in the context of e-commerce mechanisms, where incentive structures are crucial in guiding the behavior of various agents [35,36].

### 5.2. Simulation of Sincere Buyer Incentive on E-Commerce Platform

**Figure 6:**
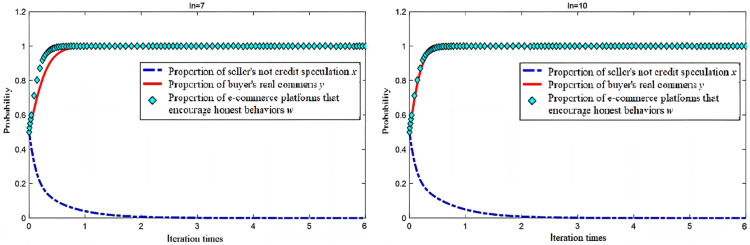
Simulation results with different In.

We analyzed the influence of blockchain incentives for buyers to make real comments during the evolutionary game process. With the other parameters unchanged, the e-commerce platform’s incentives to honest buyers (In) are assigned as 7 and 10, respectively, and the simulation results of the evolution of the replication dynamic equations over time are shown in Figure 6.

**Figure 7:**
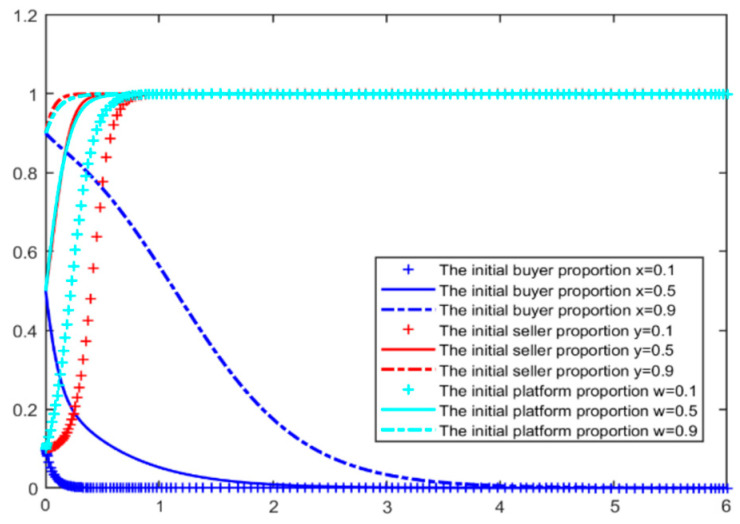
Evolution simulation results when In = 10.

In the process of system evolution toward a stable point, with the other conditions unchanged, e-commerce platforms can increase the incentives for buyers who make real comments to accelerate the evolution of buyers’ selection of real comments. This incentive mechanism helps to reduce the system’s informational entropy by promoting more predictable and honest behavior among buyers. As shown in Figure 6, with the increase in buyer incentives (In), the probability of buyers choosing to make real comments increases, reflecting a decrease in behavioral uncertainty. However, increasing incentives for sincere buyers has a less pronounced impact on sellers. As shown in Figure 7, under a high level of buyer incentives, a higher initial proportion of honest buyers significantly slows the decline rate of the proportion of honest sellers. While buyer incentives may not directly impact the proportion of sellers’ honesty, a more positive buyer response can reduce the overall system entropy by enabling sellers to reach a stable equilibrium faster, promoting more orderly and efficient interactions between the participants. Similar dynamics have been observed in blockchain-based e-commerce mechanisms, where incentives play a key role in aligning the behaviors of all participants and reducing disorder [36].

## 6. Conclusions

### 6.1. Scientific and Practical Implications

This study proposes a blockchain-based real comment mechanism, introducing innovative incentives for honest sellers and constructing a tripartite evolutionary game model involving sellers, buyers, and e-commerce platforms. By analyzing the decision-making processes of these three parties, and using numerical simulations, the factors influencing their strategic choices were examined. The following key conclusions are drawn as follows:

(1) Through an analysis of the limitations of the current comment mechanisms on mainstream e-commerce platforms, this study introduces blockchain technology by incorporating its features such as “timestamping”, smart contracts, consensus mechanisms, and “tokens.” The proposed mechanism not only enhances transparency and accountability but also reduces systemic entropy. By introducing these features, the mechanism encourages more predictable and honest interactions between buyers and sellers. Specifically, the use of tokens creates an effective incentive structure that motivates users to engage in the review process honestly, thereby enhancing the reliability and integrity of the feedback system.
(2) The simulation analysis provides compelling evidence that the blockchain-based real comment mechanism is highly effective in motivating real comments and promoting honest behavior among both buyers and sellers. Key findings from the simulation include the following:
  ① Seller Behavior: The blockchain incentive mechanism significantly reduces the disorder caused by seller dishonesty, leading sellers to evolve away from “credit speculation” strategies. As a result, sellers are more likely to adopt honest practices, which contribute to a more transparent and equitable marketplace.
  ② Buyer Behavior: The introduction of token rewards and penalties for fake reviews plays a crucial role in incentivizing buyers to provide genuine feedback, thereby helping to stabilize the system and reduce informational entropy. This creates a more predictable and reliable environment for buyers and sellers alike.
  ③ Marketplace Stability: By reducing the negative experiences caused by speculative or dishonest sellers, the mechanism encourages buyers to make real comments, which ultimately promotes a more orderly, transparent, and trustworthy marketplace.

### 6.2. Limitation and Future Research

Despite these findings, there are limitations in this study that warrant further exploration. First, while this study focuses on incentivizing buyers within a blockchain-based comment mechanism, future research should explore mechanisms that cover all three parties—sellers, buyers, and platforms. A more comprehensive approach would ensure that systemic entropy is fully optimized, promoting a balanced ecosystem where each stakeholder is incentivized to behave honestly. Integrating the roles of platforms in facilitating trust and transparency could further enhance the overall effectiveness of blockchain in reducing fake reviews. Second, certain factors were simplified in or omitted from this study for the sake of modeling clarity. However, in reality, consumers’ perceptions of the credibility of fake reviews are highly dynamic and may vary depending on factors such as product category, seller reputation, or review volume. These perceptions exhibit multimodal and nonlinear effects on product sales and buyer behavior. Future research could explore how the different perceptions of review credibility evolve over time and how these variations influence the effectiveness of blockchain incentive mechanisms. Understanding these nuances will help refine the design of blockchain-based systems to ensure that they are adaptable to changing consumer attitudes.

## Figures and Tables

**Table 1:** Parameters of the tripartite evolutionary game model.

Symbol	Description	Symbol	Description
H	The cost of operating with integrity on the part of the seller	Th	Negative experiences brought to buyers by threats, harassment, etc. of speculative sellers
En	The incentive from e-commerce platforms to buyers who make honest comments	T_2_	The cost of making a fake comment by a buyer
En_2_	The incentive from e-commerce platforms to sellers who operate in good faith	F	The income of buyers who make fake reviews, i.e., positive reviews and cashback, swiping, etc.
C_1_	The cost of the seller’s speculation	r	The income coefficient of praise cashback, swiping orders, etc.
C_2_	The excess cost to the seller when the buyer chooses the real comment	α	The probability that a buyer making a fake comment will be verified by the platform
GI	The income of the seller’s speculation business	N	Penalties for making fake comments after verification, other possible losses
ψ	The probability that the seller’s letter will be verified	Pr	The seller’s income from honest operation
λ	The penalty coefficient of the platform for speculating sellers	O_1_	The cost of detecting fake comments on the platform, including the manpower, material funds.
P	The penalties brought about by the verification of the seller’s speculation and a series of losses	O_2_	The total cost used for blockchain incentives
T	The cost of a buyer making a real comment	I	The benefits of stimulating real comments
In	Blockchain incentives for buyers to make real comments	L	Losses caused by disincentive to real comment
E	The psychological satisfaction of truthful comments		

**Table 2:** Tripartite revenue matrix based on blockchain.

Buyer	Seller	
	(X) Not Credit Speculation A1	(1 − X) Credit Speculation A2
(Y) real comments B1	Pr + En_2_ − H, E + En + In − T, I − O_1_ − O_2_	GI − C_1_ − C2 − ψ × λP, E + En + In − T − Th, I − O_1_ − O_2_
(1 − Y) fake comments B2	Pr + En_2_ − H, rF − T_2_ − αΝ, I − O_1_ − O_2_	GI − C_1_ − ψ × λP, rF − T_2_ − αΝ, I − O_1_ − O_2_
	(W) encourages honest behaviors Q1	
(Y) real comments B1	Pr − H, E + In − T, −L − O_1_	GI − C_1_ − C_2_ − ψ × λP, E + In − T − Th, −L − O_1_
(1 − Y) fake comments B2	Pr − H, rF − T_2_ − αΝ, −L − O1	GI − C_1_ − ψ × λP, rF − T_2_ − αΝ, −L − O_1_
	(1 − W) does not encourage honest behaviors Q2	

**Table 3:** Jacobian eigenvalues by using the real comment mechanism based on blockchain.

Equilibrium	Eigenvalue λ1	Eigenvalue λ2	Eigenvalue λ3
J_1_(0, 0, 0)	(Pr − H) − (GI − C1 − ψ × λP)	(E + In − T − Th) − [rF − T2 − αN]	(I − O2) − (−L)
J_2_(1, 0, 0)	(GI − C1 − ψ × λP) − (Pr − H)	(E + In − T) − [rF − T2 − αN]	(I − O2) − (−L)
J_3_(0, 1, 1)	(Pr − H) − (GI − C1 − C2 − ψ × λP)	[rF − T2 − αN] − (E + In − T − Th)	(I − O2) − (−L)
J_4_(0, 0, 1)	(En2 + Pr − H) − (GI − C1 − ψ × λP)	(En + E + In − T − Th) − [rF − T2 − αN]	(−L) − (I − O2)
J_5_(1, 1, 0)	(GI − C1 − C2 − ψ × λP) − (Pr − H)	[rF − T2 − αN] − (E + In − T)	(I − O2) − (−L)
J_6_(1, 0, 1)	(GI − C1 − ψ × λP) − (En2 + Pr − H)	(En + In + E − T) − [rF − T2 − αN]	(−L) − (I − O2)
J_7_(0, 1, 1)	(En2 + Pr-H) − (GI − C1 − C2 − ψ × λP)	[rF − T2 − αN] − (En + E + In − T − Th)	(−L) − (I − O2)
J_8_(1, 1, 1)	(GI − C1 − C2 − ψ × λP) − (En2 + Pr − H)	[rF − T2 − αN] − (En + E + In − T)	(−L) − (I − O2)

**Table 4:** Parameter setting.

Parameter	En2	Pr-H	GI	C1	C2	φ	En	Th	E
Value	10	40	100	20	6	0.74	3	4	2
Parameter	rF	T2	T	α	N	In	I	O2	L
Value	12	5	2	0.82	8	3	200	230	40

## Data Availability

The original contributions presented in the study are included in the article, further inquiries can be directed to the corresponding author.
